# Salivary Biomarkers of Inflammasome Activation in Unstable Periodontitis: A Case–Control Study

**DOI:** 10.1055/s-0045-1806931

**Published:** 2025-04-23

**Authors:** Zainab Mosa Hassan, Hadeel Mazin Akram

**Affiliations:** 1 Department of Periodontics, College of Dentistry, University of Baghdad, Baghdad, Iraq; 2 Department of Periodontics, College of Dentistry, University of Baghdad, Baghdad, Iraq

**Keywords:** periodontitis, inflammasomes, caspase, saliva, interleukin

## Abstract

**Objectives:**

The objective of this study was to investigate the complex network of inflammasome-related biomarkers (NOD-like receptor thermal protein domain associated protein 3 [NLRP3], caspase-1, interleukin [IL]-1β, IL-18, and IL-37) in unstable periodontitis by examining the salivary concentrations of these specific biomarkers and correlating them with periodontal parameters.

**Materials and Methods:**

The design of this study was an observational case–control study. A salivary sample was collected from periodontally healthy patients ( *n* = 40) and unstable periodontitis patients ( *n* = 40). Full-mouth clinical periodontal parameters were recorded (plaque index, bleeding on probing, periodontal pocket depth, and clinical attachment loss). Enzyme-linked immunosorbent assay analyzed NLRP3, caspase-1, IL-1β, IL-18, and IL-37 salivary levels.

**Statistical Analysis:**

The normality of the data was tested using the Shapiro–Wilk test. Mean, standard deviation, and percentages were used for data description. An independent sample *t* -test, Mann–Whitney *U* test, and chi-square test were used to compare the two groups with a *p* -value of < 0.05. Spearman's correlation analysis was conducted to examine the relationships between variables.

**Results:**

In saliva samples, NLRP3, caspase-1, IL-1β, and IL-18 were the highest in the periodontitis group ( *p* < 0.005), while IL-37 was highest in the healthy group ( *p* < 0.005). There was significant ( *p* < 0.012) negative weak correlation (−0.395) between IL-37 and IL-1β, and significant ( *p* < 0.003) negative moderate correlation (−0.455) between IL-37 and IL-18 in the healthy group. A significant (0.031) positive weak correlation (0.342) was found between the salivary IL-37 and NLRP3, and a significant ( *p* < 0.001) negative moderate correlation (−0.508) was found between salivary IL-37 and IL-1β, in the periodontitis group.

**Conclusion:**

The NLRP3 inflammasomes and their cytokines (caspase-1, IL-1β, and IL-18) significantly promote periodontal inflammation and tissue destruction. In contrast, IL-37 acts as an anti-inflammatory cytokine, inhibiting the activity of the NLRP3 inflammasome and reducing excessive inflammation. This interplay highlights the potential of targeting NLRP3 and enhancing IL-37 as a therapeutic approach for the treatment of periodontal disease.

## Introduction

Periodontitis is a chronic inflammatory disorder affecting the gingiva and adjacent periodontal tissues (the bone, connective tissue, adjacent oral mucosa, periodontal ligament [PDL], and gingiva). 1 2 Bacterial biofilms form on dental surfaces and provoke inflammatory reaction. 3 From 2011 to 2020, the prevalence of periodontitis among dentate adults was around 62%, with severe periodontitis affecting 23.6% of this population. This indicates an abnormally elevated prevalence of periodontitis. 4

**Fig. 1:**
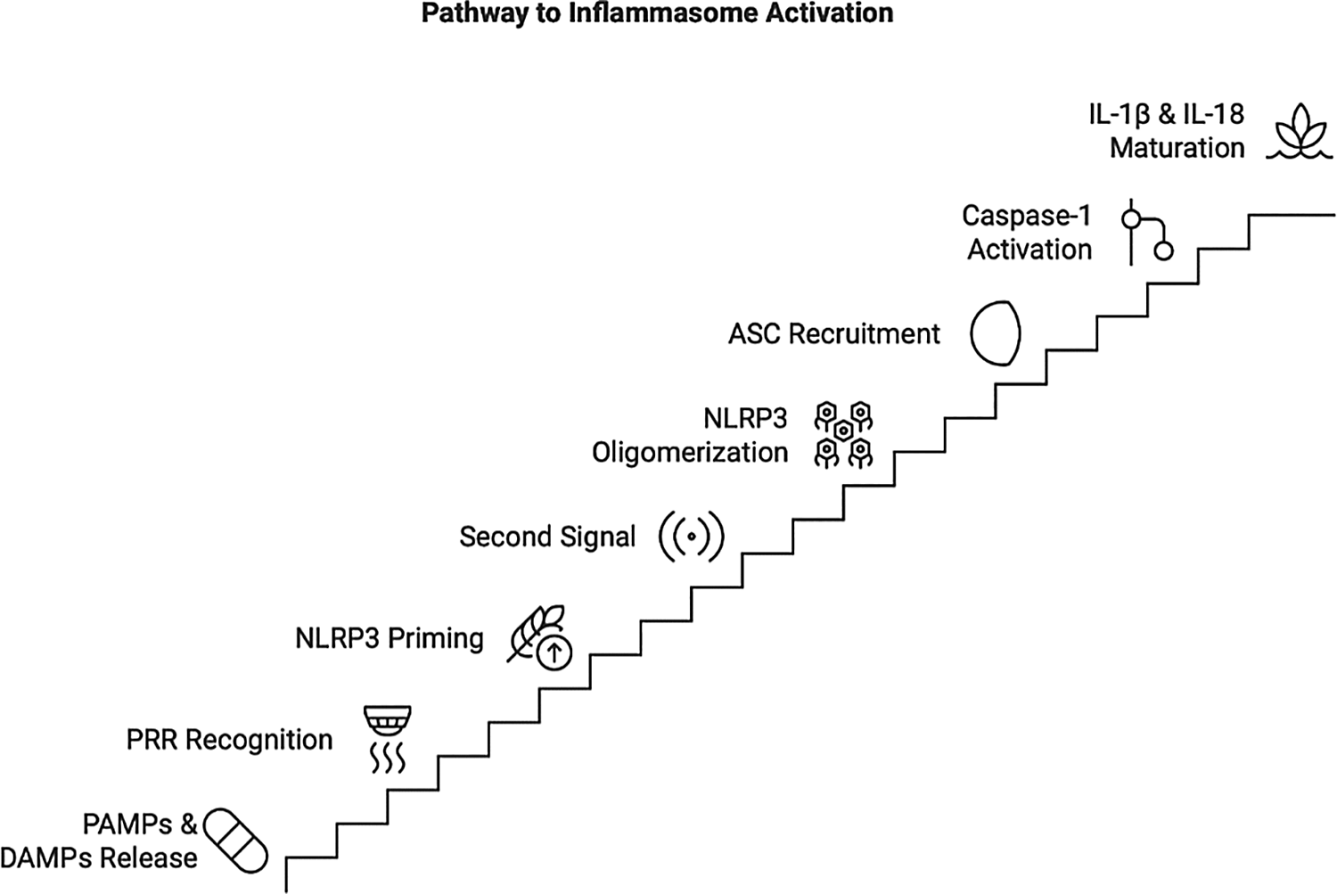
The pathway of the NOD-like receptor thermal protein domain associated protein 3 (NLRP3) inflammasome activation.

Inflammasomes are molecular signaling complexes that form in response to a threat. 5 They are intracellular pattern recognition receptors that activate upon the recognition of several signals. 6 Inflammasomes represent a component of the innate immune response that activates inflammatory caspases. 7 In the initial phase of host response, different pathogen-associated molecular patterns (PAMPs) and host-derived danger-associated molecular patterns (DAMPs) initiate inflammasome production and the release of critical inflammatory cytokines. 8 The NOD-like receptor thermal protein domain associated protein 3 (NLRP3) inflammasome is a multimeric complex composed of 1- (NLRP3), 2-apoptosis-associated speck-like protein including a caspase activation and recruitment domain (CARD) (ASC), and 3-pro-caspase-1 (pro-Casp-1). 9 10 Recruitment of the adaptor protein ASC and pro-Casp-1 to the inflammasome results in the autoproteolytic activation of pro-Casp-1 11 ( Fig. 1 ).

Fig. 1 shows the canonical inflammasome activation that is dependent on the formation of a multiprotein complex that detects danger signals and recruits pro-Casp-1, thereby facilitating the maturation of pro-interleukin (IL)-1β and pro-IL-18 into their active forms, IL-1β and IL-18, respectively, and their subsequent release. 12 The activation of the NLRP3 inflammasome is established to necessitate two consecutive phases: the priming phase and the assembly phase. 13 The nucleotide-binding regions and a leucine-rich repeat (NLR) family is distinguished by its tripartite structure. All NLRs possess a CARD or pyrin domain (PYD) at the N-terminal, a central nucleotide-binding oligomerization domain (NACHT) and a C-terminal region characterized by leucine-rich repeats, which facilitate interactions with other proteins and enhance inflammasome formation. 14 The activation of the inflammasome and pyroptosis constitute an evolutionarily conserved mechanism for pathogen defense, observed in diverse cellular types and tissues, including macrophages, monocytes, periodontal tissues, osteoclasts, and osteoblasts. 15

The IL-1β, a member of the IL-1 cytokine family, is an essential proinflammatory cytokine released by host cells in the inflammatory process of periodontal disease. 16 IL-1β secreted by macrophages not only recruits immune cells to enhance the inflammatory response but also induces collagen degradation by elevating matrix metalloproteinase secretion and facilitates receptor activator of nuclear factor kappa B ligand (RANKL)-mediated osteoclastogenesis, ultimately leading to bone resorption in periodontitis. 17 IL-1β is higher in serum, saliva, and gingival crevicular fluid of patients with periodontitis. 18

The IL-18, a member of the IL-1 cytokine family, stimulates the upregulation of other proinflammatory cytokines (tumor necrosis factor α [TNF-α], IL-1β, IL-6), perpetuating a detrimental cycle that contributes to inflammation and tissue destruction in periodontal tissue. 19

IL-37 is also a member of the IL-1 cytokine family. Recent studies have identified IL-37 as a bifunctional cytokine demonstrating significant immunosuppressive and anti-inflammatory properties. 20 It may either penetrate the nucleus directly within the cell to perform its function or be secreted extracellularly to interact with the membrane receptors of itself or adjacent cells. Evidence has established that IL-37 can diminish and suppress immune responses in inflammatory and autoimmune illnesses, reducing tissue damage. 21 Research indicates that IL-37 might regulate the expression of inflammatory cytokines and exert an anti-inflammatory effect by regulating the NLRP3 inflammasome. 22

The relationship among these biomarkers is based on the potential sequence of events that may arise within the context of periodontitis. 23 Understanding the equilibrium among these cytokines is essential, as their respective and collective impacts and their correlation with the progression of periodontitis. 24 This may provide critical insights into the fundamental causes of disease severity, treatment efficacy, and the investigation of novel therapeutic techniques that seek to target specific components within this intricate cytokine network. 25 This study investigates the complex network of inflammatory mediators by examining the salivary concentrations of five specific biomarkers: NLRP3, Casp-1, IL-1β, IL-18, and IL-37.

## Materials and Methods

### Study Settings

This observational case–control research study was conducted at the University of Al-Muthannah College of Dentistry and the Specialized Dentistry Centre in Al-Muthannah, Iraq.

Eighty subjects were recruited (mean age 35.39 ± 10.30 years; 1:1 female and male ratio) from the middle of November 2023 to August 2024. The subjects were divided into healthy periodontium ( *n* = 40) and unstable periodontitis ( *n* = 40) based on the 2017 classification of the periodontal disease and condition. 26

### Sample Size Calculating

The study was designed to detect a statistically significant difference between the two groups with a 95% power and a 5% α margin of error. The sample size was calculated using Nlrp3 (Isola et al, 2022), 27 yielding a sample size = 80.

By using this equation:



*n* = Number of samples that we need to find out

*r* = Control to cases ratio

*z*_ 1– *β*_ = It is the desired power

*Z*_ 1– *α* /2_ = Critical value and a standard value for the corresponding level of confidence

*σ* = standard deviation (SD)

*d* = effect size

Then, the sample was divided equally into two groups of same size. Those groups were the unstable periodontitis group, *n* = 40, and the healthy control group, *n* = 40.

### Study Population

Subjects included in this study were:

1. Healthy group: periodontally healthy

The clinical criteria for healthy periodontium on intact gingiva are: Bleeding on probing (BOP) < 10%, periodontal pocket depth (PPD) ≤ 3 mm, and no clinical or radiographical bone loss. 26

2. Periodontitis group: Patients had unstable generalized periodontitis (≥ 30% of teeth involved)

-The unstable status is defined as:

(a) All sites of the patient's present persistent PPD ≥ 4 mm
(b) Equal to 4 mm with BOP
(c) Full mouth BOP > 10%

Periodontitis groups were defined as 28 :

1. Interdental clinical attachment loss (CAL) is detectable at ≥ 2 nonadjacent teeth, or
2. Buccal or oral CAL ≥ 3 mm with pocketing > 3 mm is detectable at ≥ 2 teeth.

### Inclusion and Exclusion Criteria

The study participants were 18 years of age or older, of both sexes, possessed a minimum of 20 teeth, and had no history of systemic diseases.

The study excluded several categories of participants to maintain the integrity of the results. Individuals with systemic illnesses, active dental caries, or oral ulcers were not eligible for inclusion. Similarly, those who had undergone periodontal treatment or taken anti-inflammatory drugs or antibiotics within the preceding 3 months were excluded. Patients with orthodontic appliances or fixed/removable prostheses were also ineligible. The exclusion criteria extended to pregnant or lactating women, as well as those using oral contraceptives. Smokers and alcohol users were likewise omitted from the study population. It is important to note that participation in the study was voluntary, and individuals who expressed unwillingness to participate were not included in the research. These strict, stringent exclusion criteria were implemented to minimize confounding factors and ensure the reliability of the study outcomes.

### Ethical Approval Statement

The College of Dentistry, University of Baghdad's Ethics Committee approved the protocol (Reference number: 884; Project number: 884623; Date 3-12-2023). The study followed the Declaration of Helsinki in 2008. 29 Before participating in the study, all participants were asked to sign an informed consent form that provided all information describing the study's purposes and aims.

### Reliability Analysis

Before the start of the study, the investigator's performance in accurately documenting the clinical periodontal parameters (BOP, CAL, PPD) was assessed through interexaminer and intraexaminer sessions. Sessions were performed on randomly chosen patients, and the interclass correlation coefficient 30 was > 0.75.

### Salivary Collection Procedure

Participants were asked to rinse their mouths with water, and unstimulated whole saliva was collected from the patients before clinical evaluation by the passive drooling method. 31 Patients were asked to accumulate the saliva on the floor of their mouth for 5 minutes and then expectorate into a plastic cup. Note that 3 mL of the collected saliva was transferred into the test tubes to standardize the volume collected for each patient. The samples were centrifuged to remove the cell debris at 4,000 × *g* at 40°C for 10 minutes. Moreover, the supernatants were stored at −20°C until they were analyzed using enzyme-linked immunosorbent assay (ELISA). 32

### Periodontal Examination

A full periodontal examination was conducted, by the assessment of plaque index (PI) 33 on four surfaces, BOP, PPD, and CAL on six surfaces of all participants' dentition. The researchers performed the examination using a University of North Carolina probe (a calibrated periodontal probe). The six surfaces examined per tooth were mesiobuccal, distobuccal, mid-buccal, mesiolingual, distolingual, and mid-lingual, while the four surfaces assessed for PI were buccal, distal, mesial, and lingual. The diagnosis was confirmed to differentiate unstable periodontitis from other conditions.

### Laboratory Analysis of Salivary Biomarkers

Samples were thawed and centrifuged at 1,000 revolutions per minute for 1 minute at 4°C, and 100 µm of the supernatant was collected to analyze protein biomarkers. Salivary levels of NLRP3, Casp-1, IL-1β, IL-18, and IL-37 were measured using the ELISA kit.

1. NLRP3: USCN ELISA kit product no. SEC034Hu (headquartered in Houston, United States)
  -Lower limit of detection: < 0.115 ng/mL and the detection range: 0.312–20 ng/mL
2. Casp-1: Elabscience ELISA KIT product no. E-EL-H0016 (headquartered in Houston, United States)
  -Sensitivity: 46.88 pg/mL and the detection range: 78.13–5000 pg/mL
3. IL-1β: Human IL-1β ELISA Kit from Elabscience, product no. E-EL-H0149 (headquartered in Houston, United States)
  -Sensitivity: 4.69 pg/mL and the detection range: 7.81–500 pg/mL
4. IL-18: Human IL-18 ELISA Kit, product no. E-EL-H0253 H0149 (headquartered in Houston, United States)
  -Sensitivity: 9.38 pg/mL and the detection range: 15.63–1000 pg/mL
5. IL-37: Human IL-37 ELISA Kit, product no. E-EL-H2571 (headquartered in Houston, United States)
  -Sensitivity: 9.38 pg/mL and the detection range: 15.63–1000 pg/mL

### Statistical Analysis

For continuous variables, central tendency and dispersion were quantified using mean values and SD. The Shapiro–Wilk test was applied to evaluate the normality of data distribution. Intergroup comparisons were performed using an independent sample *t* -test, Mann–Whitney *U* test, and chi-square test. Within each group, Spearman's correlation analysis was conducted to examine the relationships between variables. Positive and negative predictive values were determined through contingency table analysis. Statistical significance was defined as *p* < 0.05.

## Result

**Fig. 2:**
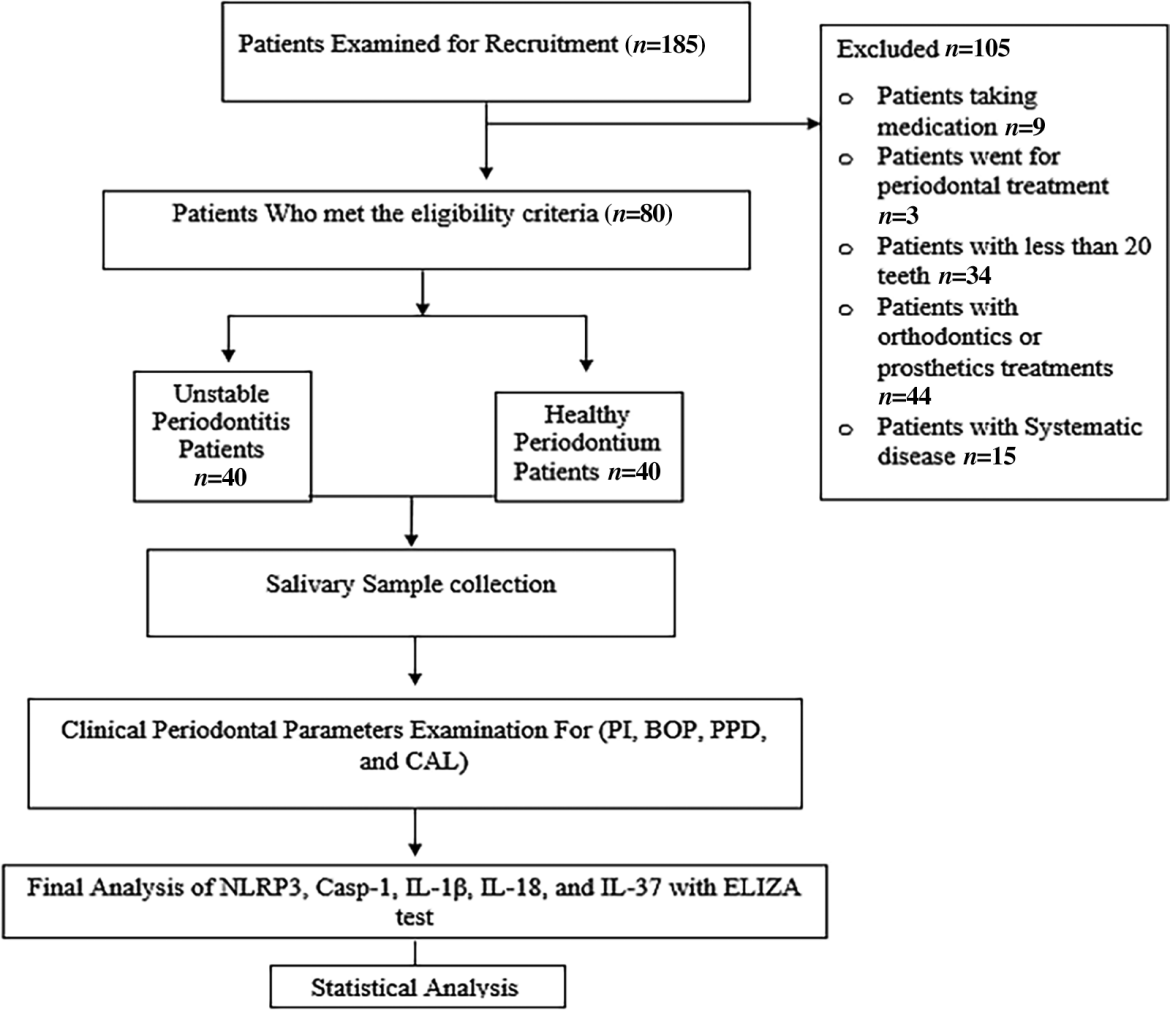
Flowchart of the study.

The total number examined for eligibility criteria to participate in this study was *n* = 185. Only 80 participants met the inclusion criteria; 40 of them had healthy periodontium and included in the healthy group. The rest were excluded due to a variety of exclusion criteria, as shown in Fig. 2

Table 1 illustrates age characteristics, sex distribution, and the number of missing teeth. The average age was 35.39 ± 10.30 years, ranging between 18 and 63 years, and there was no significant difference between the healthy and periodontitis groups ( *p* = 0.077). In the healthy group, the mean age was 33.35 ± 6.84 years, ranging from 23 to 45 years, while in the periodontitis group, the mean was 37.43 ± 12.64 years, ranging between 18 and 63 years. Distribution according to sex showed that males ( *n* = 40) represented 50%, and females ( *n* = 40) represented 50% of the total sample. There was no significant difference in the proportions of males and females between both groups ( *p* = 0.074) ( Table 1 ).

**Table 1:** Study population demographic characters

Variables	Group	*N*	Min. (y)	Max. (y)	Mean (y)	SD	*p* -Value
Age (y)	Healthy	40	23	45	33.35	± 6.84	0.077 a
	Periodontitis	40	18	63	37.43	± 12.64	
	Total	80	18	63	35.39	± 10.30	
Sex	**Group**	***N***	**Male** ***N*** **(%)**	**Female** ***N*** **(%)**	**Total** ***N*** **(%)**		***p*** **-Value**
	Healthy	40	16 40)	24 (60)	40 (50)		0.074
	Periodontitis	40	24 (60)	16 (40)	40 (50)		
	Total	80	40 (50)	40 (50)	80 (100)		
No. of missing teeth	**Group**	**Min.**	**Max.**	**Total**	**Mean ± SD**		***p*** **-Value**
	Healthy	0	7	51	1.28 ± 1.77		< 0.001 a
	Periodontitis	0	8	162	4.05 ± 3.15		
	Total	0	8	213	4.63 ± 17.93		

Abbreviations: %, frequency; Max, maximum; Min, minimum; SD, standard deviation.

a Mann–Whitney *U* test.

There was a significantly higher number of missing teeth in the periodontitis group compared with the healthy group ( *p* < 0.001). The total number of all missing teeth in both groups was 213, with a mean of 4.63 ± 17.93 teeth. The lowest was in the healthy group, 51 teeth, and the mean was 1.28 ± 1.77. In periodontitis, the total number of missing teeth was 162, and the mean was 4.05 ± 3.15 ( Table 1 ).

### Analysis of Clinical Periodontal Parameters and Salivary Biomarkers

#### The Periodontal Parameters

The mean percentage of the PI was significantly higher ( *p* < 0.001) in periodontitis (93.2% ± 0.17) compared with the healthy group (10.3% ± 0.06), and the mean percentage of BOP was also significantly higher ( *p* < 0.001) in the periodontitis group (62.5% ± 0.23) compared with the healthy group (5.8% ± 0.03). The mean value of PPD was 4.46 ± 0.51 mm, and the CAL was 3.7 ± 1.41 mm in the periodontitis group ( Table 2 ).

**Table 2:** Descriptive and comparison statistics of clinical parameters and salivary biomarkers

Variable	Group	*N*	Mean	SD	Min.	Max.	*p* -Value
PI%	Healthy	40	10.3%	± 0.061	0.9%	20%	< 0.001 a
	Periodontitis	40	93.2%	± 0.168	44%	100%	
BOP%	Healthy	40	5.8%	± 0.027	0.7%	9.8%	< 0.001 a
	Periodontitis	40	62.5%	± 0.237	27%	100%	
PPD (mm)	Periodontitis	40	4.46	± 0.513	4.000	5.880	
CAL (mm)	Periodontitis	40	3.697	± 1.405	1.390	10.390	
NLRP3 (ng/mL)	Healthy	40	7.52	± 0.955	5.330	9.400	< 0.001 a
	Periodontitis	40	22.350	± 2.360	15.830	26.330	
Casp-1 (pg/mL)	Healthy	40	1239.24	± 265.414	769.150	1796.520	< 0.001 a
	Periodontitis	40	2903.91	± 798.047	1672.020	4410.100	
IL-1β (pg/mL)	Healthy	40	64.555	± 27.75	10.410	113.990	< 0.001 b
	Periodontitis	40	333.253	± 56.16	202.110	463.450	
IL-18 (pg/mL)	Healthy	40	238.184	± 73.354	80.410	369.980	< 0.001 b
	Periodontitis	40	630.52	± 110.5	380.830	808.810	
IL-37 (pg/mL)	Healthy	40	554.726	± 152.275	312.925	1000.000	< 0.001 a
	Periodontitis	40	281.926	± 97.653	161.280	505.880	

Abbreviations: BOP%, bleeding on probing percentage; CAL, clinical attachment loss; Casp-1, caspase-1; IL, interleukin; Max, maximum; Min, minimum; NLRP3, NLR family pyrin domain containing 3; PI%, plaque index percentage; PPD, periodontal pocket depth.

a Mann–Whitney *U* test.

b Independent sample *t* -test.

#### The Salivary Biomarkers

The NLRP3 level was significantly higher ( *p* < 0.001) in the periodontitis group, 22.35 ± 2.36 ng/mL, compared with the healthy group, 7.51 ± 0.95 ng/mL. Similarly, the salivary Casp-1 level was significantly higher ( *p* < 0.001) in the periodontitis group, 2903.91 ± 798.04 pg/mL, compared with the healthy group, 1239.24 ± 265.41 pg/mL.

The salivary level of IL-1β in the healthy group, 64.55 ± 27.75 pg/mL, was significantly lower ( *p* < 0.001) than in the periodontitis group, 333.25 ± 56.16 pg/mL, and the salivary level of IL-18, it was significantly higher ( *p* < 0.001) in the periodontitis group, 630.52 ± 110.5 pg/mL, compared with the healthy group, 238.18 ± 73.35 pg/mL. Conversely, IL-37 was the only salivary biomarker that exhibited significantly higher levels ( *p* < 0.001) in the healthy group, 554.726 ± 152.275 pg/mL, compared with the periodontitis group, 281.926 ± 97.653 pg/mL ( Table 2 ).

### Correlation Correlations between Salivary Biomarkers and Periodontal Parameters in the Healthy Group

**Fig. 3:**
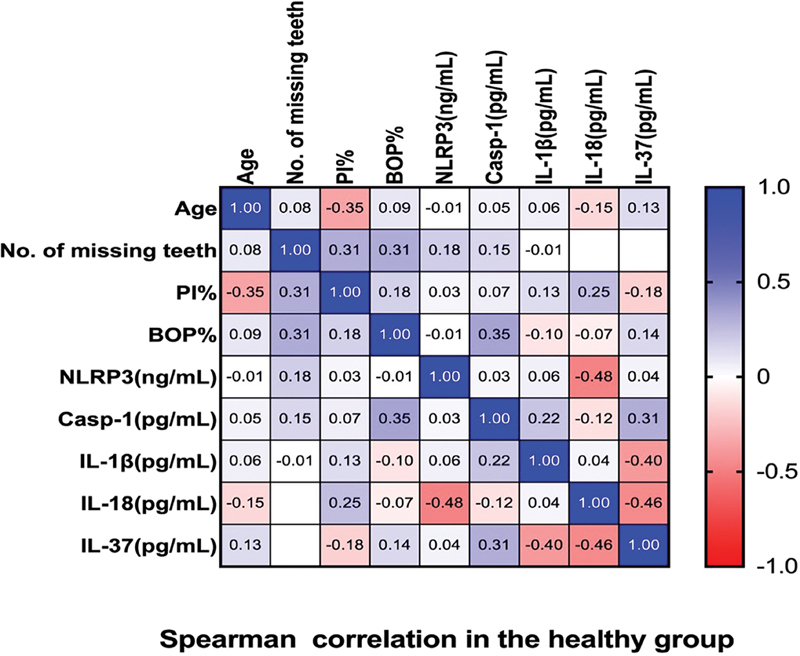
The correlation between the healthy group's periodontal parameters and salivary biomarkers.

Fig. 3 demonstrates the correlation of the healthy group between periodontal parameters and the salivary biomarkers.

**Fig. 4:**
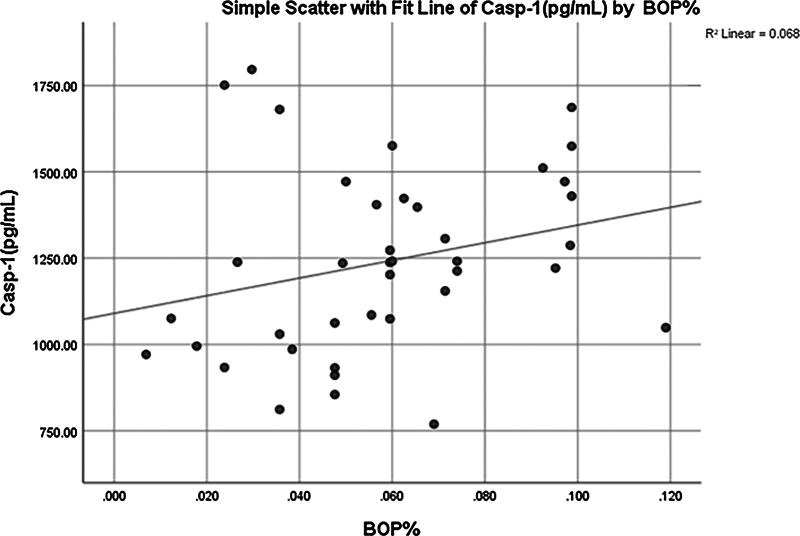
Simple linear regression between caspase-1 (Casp-1) and bleeding on probing percentage (BOP%).

**Fig. 5:**
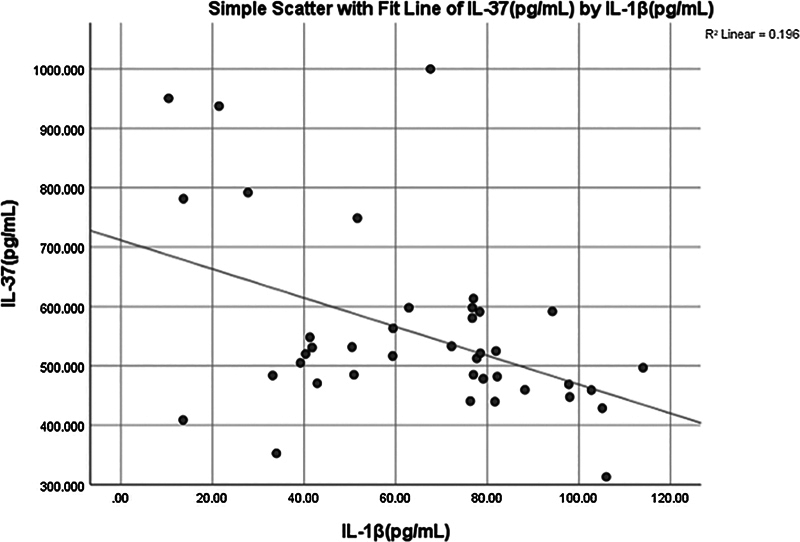
Simple linear regression between interleukin (IL)-37 and IL-1β.

**Fig. 6:**
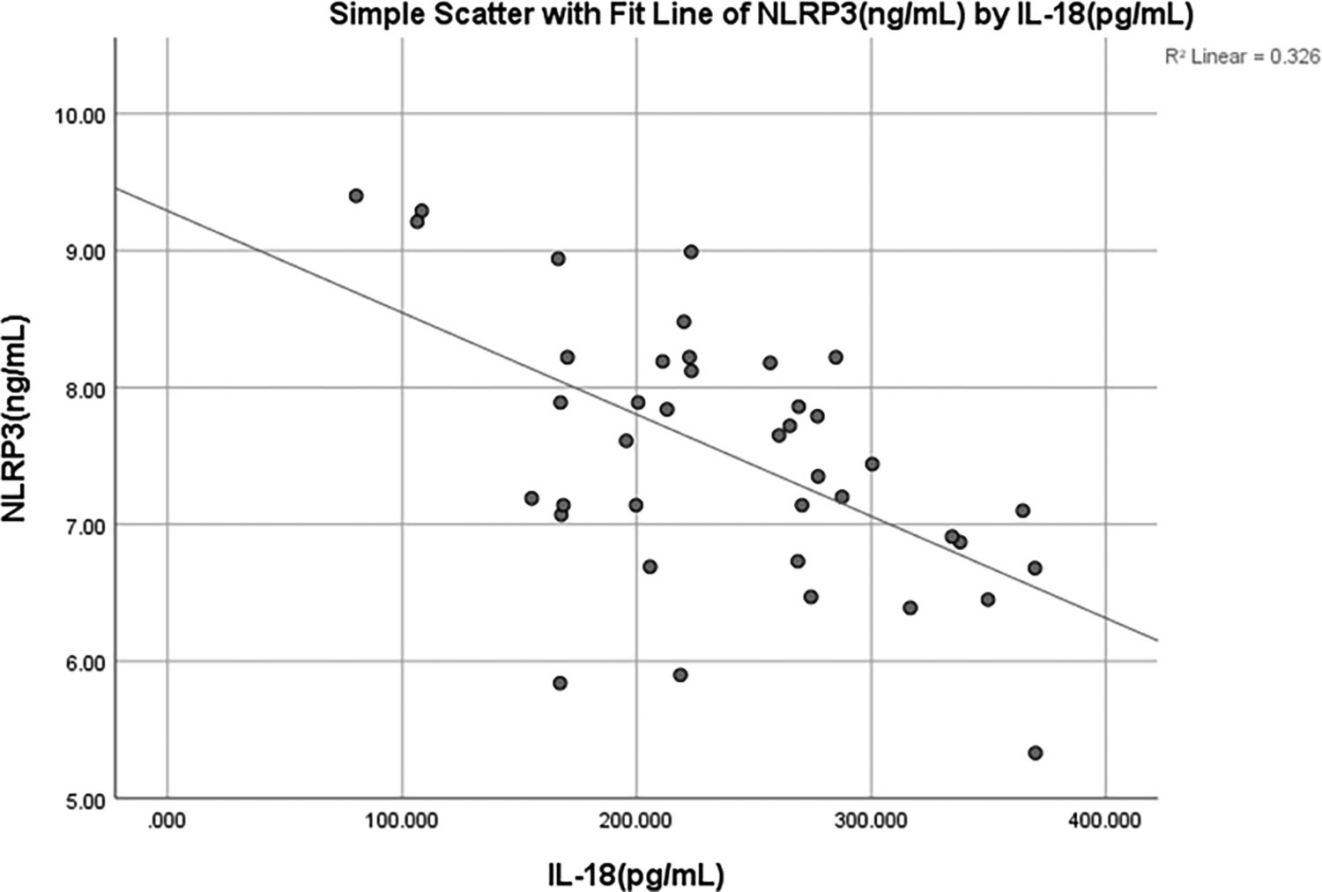
Simple linear regression between interleukin (IL)-18 (pg/mL) and NOD-like receptor thermal protein domain associated protein 3 (NLRP3).

**Fig. 7:**
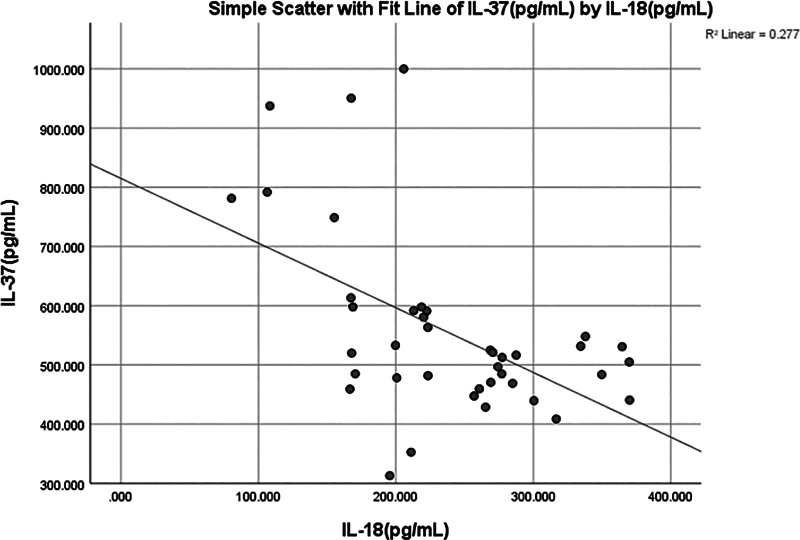
Simple linear regression between interleukin (IL)-37 (pg/mL) and IL-18 (pg/mL).

- Significant ( *p* < 0.026) positive weak correlation (0.353) was observed between Casp-1 and BOP% ( Figs. 3 and 4 ).
- Significant ( *p* < 0.012) negative weak correlation (−0.395) was observed between IL-37 and IL-1β ( Figs. 3 and 5 ).
- Significant ( *p* < 0.002) negative moderate correlation (−0.483) was observed between IL-18 and NLRP3 ( Figs. 3 and 6 ).
- Significant ( *p* < 0.003) negative moderate correlation (−0.455) was observed between IL-37 and IL-18 ( Figs. 3 and 7 ).

### Correlations between Salivary Biomarkers and Periodontal Parameters in the Periodontitis Group

**Fig. 8:**
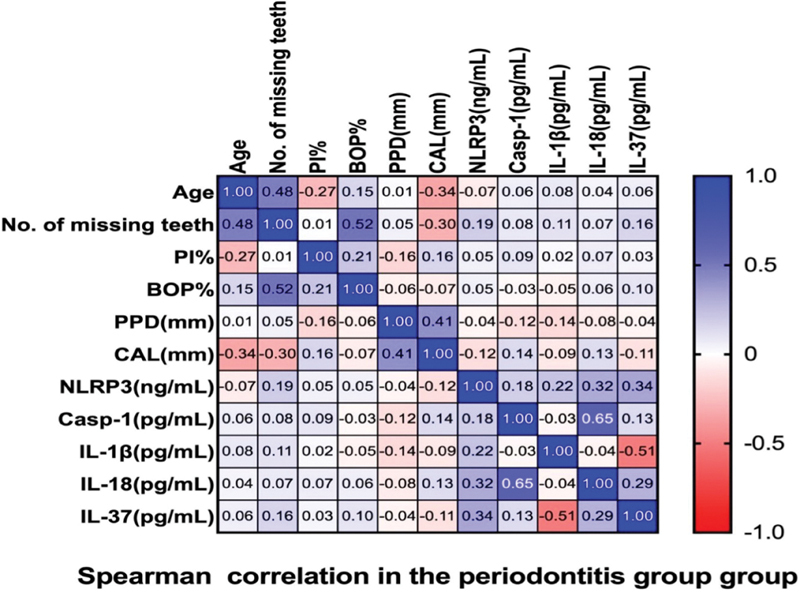
The correlation between the periodontitis group's periodontal parameters and salivary biomarkers.

Fig. 8 shows the correlations between the periodontal parameters and salivary biomarkers in the periodontitis group.

**Fig. 9:**
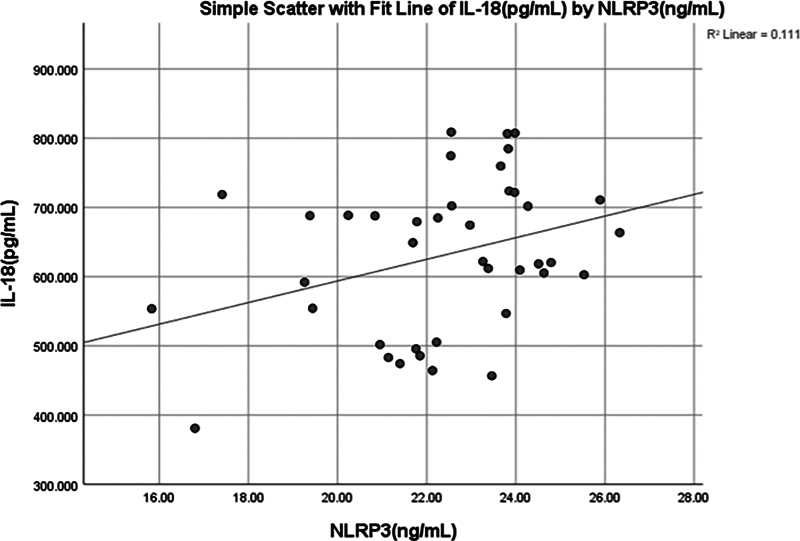
Simple linear regression between interleukin (IL)-18 (pg/mL) and NOD-like receptor thermal protein domain associated protein 3 (NLRP3) (ng/mL).

**Fig. 10:**
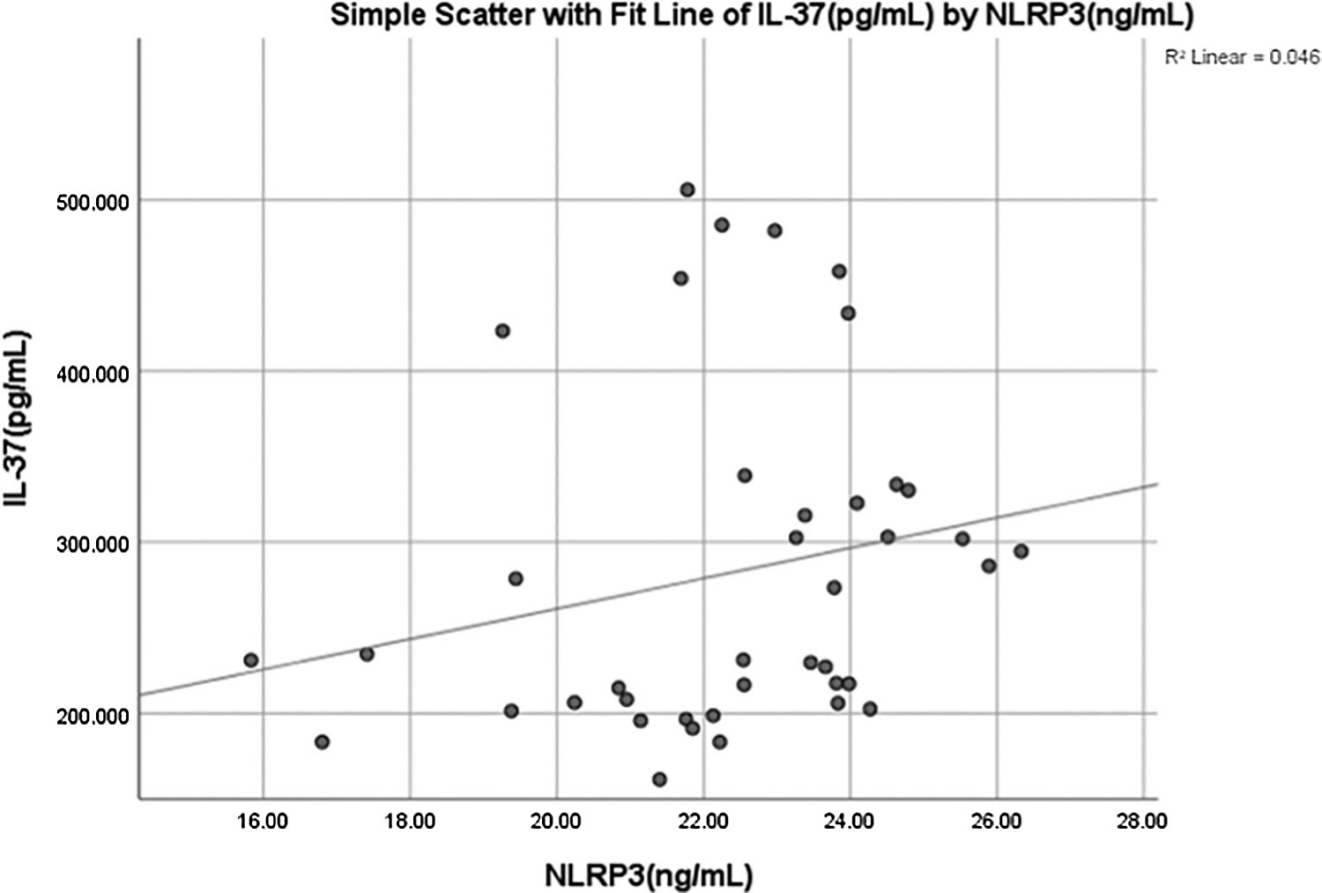
Simple linear regression between interleukin (IL)-37 (pg/mL) and NOD-like receptor thermal protein domain associated protein 3 (NLRP3) (ng/mL).

**Fig. 11:**
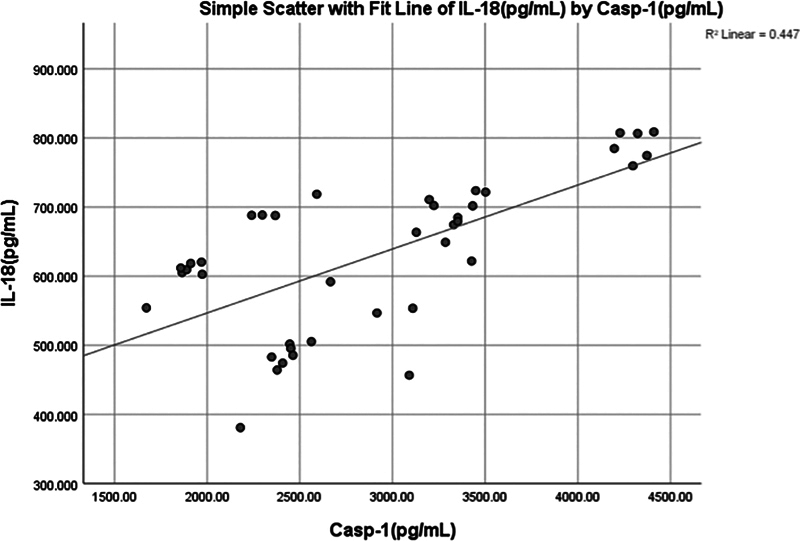
Simple linear regression between interleukin (IL)-18 (pg/mL) and caspase-1 (Casp-1) (pg/mL).

**Fig. 12:**
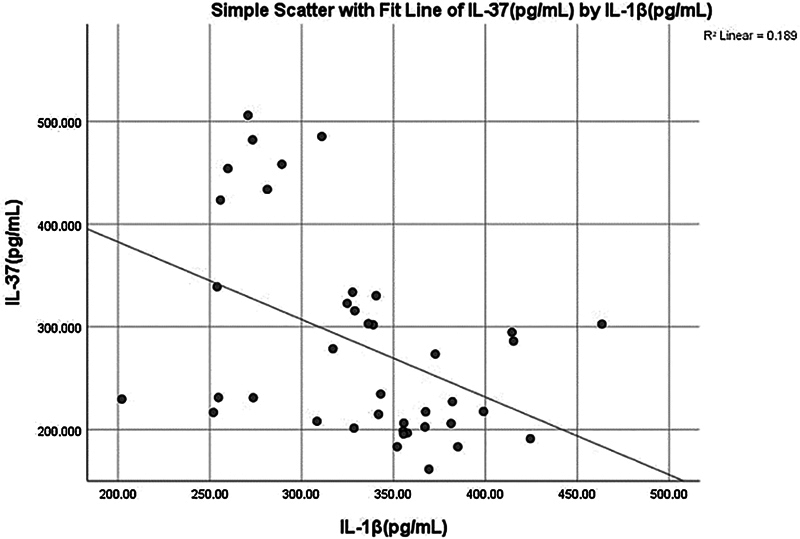
Simple linear regression between interleukin (IL)-37 (pg/mL) and IL-1β (pg/mL).

The result was:

- A significant ( *p* < 0.045) positive weak correlation (0.319) was found between salivary IL-18 and NLRP3 ( Figs. 8 and 9 ).
- A significant (0.031) positive weak correlation (0.342) was found between salivary IL-37 and NLRP3 ( Figs. 8 and 10 ).
- A significant ( *p* < 0.001) positive moderate correlation (0.654) was found between salivary IL-18 and Casp-1 ( Figs. 8 and 11 ).
- A significant ( *p* < 0.001) negative moderate correlation (−0.508) was found between salivary IL-37 and IL-1β ( Figs. 8 and 12 ).

## Discussion

In periodontium, any severe inflammatory reaction is expected to promote the resorption of supporting bones and inhibit the process of osseointegration. 34 35

The present research provides valuable insights into the role of inflammasomes and associated cytokines in the pathogenesis of periodontitis. The findings of this study demonstrated that NLRP3 inflammasome and its downstream products, including Casp-1, IL-1β, and IL-18, are significantly higher in the periodontitis patients than in the healthy group. Conversely, the IL-37, an anti-inflammatory cytokine, was significantly lower in periodontitis patients than the healthy controls. These observations suggest a complex interplay between proinflammatory (NLRP3, Casp-1, IL-1β, IL-18) and anti-inflammatory pathways (IL-37) in the progression of periodontal disease. 22 36 37 38 This makes them particularly pertinent for examining the pathogenesis and advancement of periodontitis. Examining the inflammasome pathway in periodontitis may enhance periodontal research's overall domain and better understand the interconnection between systemic inflammation and periodontal health.

Saliva was used among accessible oral fluids in this study. Saliva exhibits remarkable efficacy in identifying periodontitis and a commendable capability in detecting nonperiodontitis. 39 Saliva collection is a noninvasive and uncomplicated procedure that facilitates the acquisition of samples from several individuals without inducing discomfort. Passive drooling was chosen for saliva collection in this study since it potentially reduces bacterial contamination of the sample and other systemic mistakes linked to alternative collection methods. Furthermore, a significant amount of saliva can be obtained briefly using the drooling technique. 40 41 42

The significant elevation of salivary levels of NLRP3 inflammasome in the periodontitis group, as shown in Table 2 , indicates its role in the inflammatory process. 43 44 45 Upon activation, the NLRP3 inflammasome complex releases inflammatory cytokines. 46 These cytokines facilitate the eradication of harmful microorganisms as a defensive response. 47 Excessive amounts of inflammatory cytokines are detrimental because they play a role in collagen degradation, alveolar bone resorption, and loss of periodontal attachment. The inflammation impacts the alveolar bone between the teeth and the adjacent connective tissue. 48 NLRP3 controls bone resorption in periodontitis by facilitating osteoclast differentiation. 48 This discovery substantiates the role of NLRP3 in periodontitis.

Recent studies have also associated the overexpression and activation of the NLRP3 inflammasome with the onset of periodontitis. 49 The NLRP3 inflammasome performs distinct regulatory activities within PDL, promotes osteoclastogenesis by elevating RANKL production or reducing osteoprotegerin levels, simultaneously induces osteoblast apoptosis, increases proinflammatory cytokines in PDL fibroblasts, and regulates immune cell activities. 49 Suggesting there is a correlation between the NLRP3 and periodontitis; those findings matched the findings of this research. While other research indicated that the NLRP3 inflammasome is not significantly involved in inflammatory bone resorption. 50

The observed increase in Casp-1 levels in periodontitis patients, as depicted in Table 2 , is consistent with its role in the activation of the inflammasome, the Casp-1 cleaves pro-IL-1β and pro-IL-18 into their mature active cytokine forms. 51 Which makes the Casp-1 the primary downstream effector of all inflammasomes. 50 Activation of Casp-1 also causes rapid cell death, marked by plasma membrane rupture and the release of proinflammatory intracellular components. 52 Elevated levels of Casp-1 in saliva suggest that an active inflammation occurs in the periodontal tissues. This study agreed with a study done by Mahmood and Abbas that found the salivary levels of Casp-1 were higher in periodontitis patients than in healthy controls, indicating a correlation with the disease presence. 53 This indicates that salivary Casp-1 is involved in periodontitis. However, another study showed that the concentration of Casp-1 in saliva samples makes its measurement ineffective for detecting the presence and/or severity of periodontal disease. 36

Both IL-1β and IL-18 showed significantly higher levels in the periodontitis group than in the healthy group, consistent with other studies. 54 Upon cellular stimulation, PAMPs and DAMPs induce the assembly of inflammasomes. Pyroptosis is facilitated by the activation of Casp-1 through the NLRP3. 55 Casp-1 cleaves gasdermin family members, including gasdermin D (GSDMD), leading to cell membrane perforation by releasing its N-terminal domain. Activating Casp-1 via NLRP3 represents the typical canonical inflammasome pathway of pyroptosis. This will culminate in the cleavage of GSDMD. 56 The mechanisms of pyroptosis fundamentally require the activation of GSDMD, resulting in the formation of pores in the cell membrane, through which cytoplasmic chemicals, including IL-1β and IL-18, are released, triggering a vigorous inflammatory response. 57 L-1β is an essential proinflammatory cytokine predominantly released by monocytes, macrophages, and dendritic cells (DCs). IL-1β enhances vasodilation, and the chemotaxis of inflammatory cells promotes collagen degradation through the upregulation of matrix metalloproteinases and stimulates bone resorption by accelerating osteoclastogenesis. 58 The persistent activation of cytokines has been demonstrated to gradually impair the adjacent structures, including gingival tissue, PDL, and bone. Research indicates that IL-1β can promote the migration of proinflammatory cells from the bloodstream to inflamed tissues; additionally, it signals the extracellular matrix and triggers the production of other cytokines. 59 It has been proposed that additional noncanonical inflammasome pathways, including Casp-4, Casp-5, and Casp-8, may facilitate or offer alternative mechanisms for releasing IL-1β. This may explain the increasing levels of IL-1β in the periodontitis group. 60 61 A 2009 study conducted in Finland examined salivary cytokine levels to clarify periodontitis. IL-1β was present in all samples. 62 All patients with periodontitis in this study showed significantly elevated levels of IL-1β. Research indicates that targeting IL-1β or the NLRP3 inflammasome can significantly reduce bone loss associated with periodontitis. This suggests that IL-1β plays a crucial role in the development of the disease. 9

IL-18 is predominantly secreted by DCs, which stimulate the production of interferon-γ from Th1 cells and IL-17 from Th17 cells while also enhancing the release of IL-17, TNF-α, and IL-1β, so facilitating increased osteoclastogenesis and bone resorption. 63

Salivary concentrations of IL-18 were observed to be five times higher in people with periodontitis compared with healthy individuals. 64 While another study found no difference in salivary IL-18 levels between people with periodontitis and those without. 65

There was a significant positive correlation between NLRP3 and IL-18 ( Fig. 9 ) in the periodontitis group, supporting the belief that the NLRP3 inflammasome plays a pivotal role in the inflammatory cascade associated with periodontitis. IL-18, in particular, has been shown to increase the production of other proinflammatory cytokines, such as TNF-α and IL-6, and prolong the inflammatory response. 19 This could be one of the reasons for the progressive tissue destruction observed in periodontitis.

This investigation demonstrated an elevation in the concentration of IL37 in the saliva of individuals with healthy periodontium compared with those with periodontitis, which was inconsistent with a previous study that reported an increase in the salivary level of IL-37 in the periodontitis group. 66 IL-37 has been shown to suppress the production of proinflammatory cytokines and inhibit the activation of the NLRP3 inflammasome. 20 21

The role of IL-37 in suppressing the innate immune response has been demonstrated. It can be activated by Casp-1 cleavage, thereafter functioning as a cytokine via intracellular or extracellular routes. 67 Both the precursor and mature forms of IL-37 can bind to IL-18 receptor alpha (IL-18Rα). Mature IL-37 can also be associated with IL-18BP, the natural antagonist of IL-18, in an extracellular manner. 58 The anti-inflammatory efficacy of IL-37 demands IL-1R8. IL-37 needs the receptors IL-18Rα and IL-1R8 to inhibit innate immunity. 68 Upon binding to the IL-18Rα chain, IL-37 recruits TIR-8/IL-1R8/SIGIRR (Toll/IL-1 receptor/single Ig IL-1-related receptor), leading to the assembly of a triple complex on the cell surface. This results in innate and acquired immunosuppression alongside an enhancement of the anti-inflammatory pathway rather than the activation of the IL-18 pathway. 69

IL-37 serves a dual effect in suppressing IL-1β-mediated inflammation: it suppresses IL-1β production in activated macrophages. And significantly reduces ASC oligomerization, which precedes Casp-1 activation in inflammasome activity. 70 IL-37 markedly suppressed the expression of NLRP3, Casp-1, and IL-1β in periodontitis, considerably reduced the levels of proinflammatory cytokines TNF-α and IL-6, and enhanced the expression of the anti-inflammatory cytokine IL-10. These findings indicate that IL-37 effectively regulates the nuclear factor kappa-B/NLRP3 signaling pathway in periodontitis. 22 IL-37 also inhibits the production of several proinflammatory cytokines. 71 Consequently, it is reasonable to propose that other inflammatory cytokines have a significant role in the pathogenesis of periodontitis. A study revealed that upon stimulation of the innate immune system, macrophages generate both the proinflammatory cytokine IL-1β and the anti-inflammatory cytokine IL-37 via NLRP3 activation, thereby initiating a negative feedback process that reduces excessive inflammation. 72

Recent research has unveiled an intriguing disparity regarding IL-37 levels with periodontal health. One study indicates an increase in IL-37 levels in individuals diagnosed with periodontitis 73 ; conversely, another study demonstrates that healthy individuals possess higher IL-37 levels, 74 which matches the current study. In another contrast with the current study showed no significant difference in salivary IL-37 levels between the periodontitis group and the healthy group. 75 This notable contrast presents new opportunities for advancing the understanding of oral health and its complexities. This study revealed that IL-37 has no association between the clinical periodontal measures and IL-37 levels in the saliva. This may suggest that IL-37 is not a reliable marker for assessing periodontal disease (40).

The negative correlation between IL-37 and IL-1β in the periodontitis group ( Fig. 12 ) suggests that IL-37 may play a protective role against the excessive inflammation observed in periodontitis. This finding is consistent with previous studies that have highlighted the potential of IL-37 as a therapeutic target in inflammatory diseases. 22 24

The significant correlations between salivary biomarkers and clinical periodontal parameters, such as BOP% and PPD, suggest that these biomarkers could serve as potential indicators of disease activity. For instance, the positive correlation between Casp-1 and BOP% ( Fig. 4 ) in the healthy group indicates that even mild inflammation may be associated with activating the NLRP3 inflammasome. This finding underscores the importance of early intervention in periodontal disease management. 76

Many studies support the role of NLRP3, IL-1β, and IL-18 as diagnostic biomarkers. One study found that NLRP3 is a potential biomarker for periodontitis. 77 Another study combined NLRP3 and IL-18. This study suggests that IL-18 may not serve as an appropriate indicator for assessing the inflammatory status of periodontal tissues, whereas NLRP3 may be regarded as a marker of inflammation in periodontitis. 78 Although a systematic review found IL-18 has the potential to be a diagnostic biomarker for periodontitis. 19

The role of IL-1β as a strong diagnostic biomarker has been highlighted in several researches. 79 80 81

A recent study done by Mahmood and Abbas found that Casp-1 showed high sensitivity and specificity in the diagnosis of periodontitis. 53

A limited number of studies included IL-37 as a potential biomarker. A study suggests that IL-37 may serve as possible biomarkers that require additional longitudinal clinical research to ascertain their efficacy as prognostic or diagnostic indicators. 74 Another study found IL-37 as an unuseful diagnostic biomarker for periodontitis. 75

## Limitations and Future Directions

Although this study provides valuable insights into the role of inflammasomes and cytokines in periodontitis, some limitations should be acknowledged. The case–control design of this study prevents any conclusions regarding causality. Longitudinal studies are needed to assess the relationships between inflammasome activation, cytokine production, and the progression of periodontitis.

Future research should search for the potential of targeting the NLRP3 inflammasome and IL-37 as therapeutic strategies in periodontitis.

This study examined periodontitis comprehensively without delving into specific stages or grades. However, we recommend conducting further investigations to provide a more detailed assessment and tailored recommendations

Given the developing evidence of the role of the NLRP3 inflammasome in chronic inflammation, inhibitors of NLRP3 could be promising candidates for periodontal therapy. Similarly, strategies to enhance IL-37 production or activity may help restore the balance between proinflammatory and anti-inflammatory pathways in periodontitis.

## Conclusion

The NLRP3 inflammasomes, along with related cytokines (Casp-1, IL-1β, and IL-18), play an important role in promoting periodontal inflammation and tissue damage. IL-37, on the other hand, is a cytokine that has anti-inflammatory properties. It does this by suppressing the activity of the NLRP3 inflammasome, which has the effect of reducing excessive inflammation. This interaction highlights the importance of targeting NLRP3 and enhancing IL-37 as a therapeutic approach for treating periodontal disease.
